# Anatomical Characterization of the Human Structural Connectivity between the Pedunculopontine Nucleus and Globus Pallidus via Multi-Shell Multi-Tissue Tractography

**DOI:** 10.3390/medicina56090452

**Published:** 2020-09-07

**Authors:** Salvatore Bertino, Gianpaolo Antonio Basile, Giuseppe Anastasi, Alessia Bramanti, Bartolo Fonti, Filippo Cavallaro, Daniele Bruschetta, Demetrio Milardi, Alberto Cacciola

**Affiliations:** 1 Brain Mapping Lab, Department of Biomedical, Dental Sciences and Morphological and Functional Images, University of Messina, 98125 Messina, Italy; gbasile94@hotmail.it (G.A.B.); dptbiosciences@gmail.com (G.A.); dmilardi@unime.it (D.M.); 2 IRCCS Centro Neurolesi “Bonino Pulejo”, 98124 Messina, Italy; alessia.bramanti@gmail.com (A.B.); bartolo97fonti@gmail.com (B.F.); 3 Physical Rehabilitation Medicine and Sport Medicine Unit, University Hospital Policlinico “G. Martino”, 98124 Messina, Italy; fcavallaro@unime.it (F.C.); dbruschetta@unime.it (D.B.)

**Keywords:** pedunculopontine nucleus, globus pallidus, basal ganglia, tractography, structural connectivity, pallidotegmental tract, brainstem

## Abstract

*Background and objectives:* The internal (GPi) and external segments (GPe) of the globus pallidus represent key nodes in the basal ganglia system. Connections to and from pallidal segments are topographically organized, delineating limbic, associative and sensorimotor territories. The topography of pallidal afferent and efferent connections with brainstem structures has been poorly investigated. In this study we sought to characterize in-vivo connections between the globus pallidus and the pedunculopontine nucleus (PPN) via diffusion tractography. *Materials and Methods*: We employed structural and diffusion data of 100 subjects from the Human Connectome Project repository in order to reconstruct the connections between the PPN and the globus pallidus, employing higher order tractography techniques. We assessed streamline count of the reconstructed bundles and investigated spatial relations between pallidal voxels connected to the PPN and pallidal limbic, associative and sensorimotor functional territories. *Results:* We successfully reconstructed pallidotegmental tracts for the GPi and GPe in all subjects. The number of streamlines connecting the PPN with the GPi was greater than the number of those joining it with the GPe. PPN maps within pallidal segments exhibited a distinctive spatial organization, being localized in the ventromedial portion of the GPi and in the ventral-anterior portion in the GPe. Regarding their spatial relations with tractography-derived maps of pallidal functional territories, the highest value of percentage overlap was noticed between PPN maps and the associative territory. *Conclusions:* We successfully reconstructed the anatomical course of the pallidotegmental pathways and comprehensively characterized their topographical arrangement within both pallidal segments. PPM maps were localized in the ventromedial aspect of the GPi, while they occupied the anterior pole and the most ventral portion of the GPe. A better understanding of the spatial and topographical arrangement of the pallidotegmental pathways may have pathophysiological and therapeutic implications in movement disorders.

## 1. Introduction

The globus pallidus (GP) is subdivided into internal (GPi) and external segments (GPe) by the lamina medullaris interna, a thin strip of white matter [1]. The GPi and GPe play distinct roles in basal ganglia circuitry: the former is one of the output nuclei, the latter is an integrative node, receiving afferent connections from other basal ganglia structures and sending projections to both the GPi and the striatum [2]. Primates studies revealed that the main connectional systems of both pallidal segments are topographically organized, delineating limbic, associative and sensorimotor territories [3,4,5]. This peculiarity is shared across basal ganglia nuclei and it has been confirmed in human imaging studies carried out via diffusion magnetic resonance imaging (dMRI) and tractography-related techniques [6,7,8,9]. Such a topographical organization has gained increasing attention in behavioral neuroscience, as the tripartite organization matches existing evidence for motivational, cognitive and motor roles of the GP [10], as well as in functional neurosurgery for its therapeutic implications in movement disorders [11,12,13].

Despite the vast majority of human studies focused especially on striatofugal and pallidofugal pathways, connections between the GP and brainstem nuclei are widely documented [14]. Among these, ipsilateral and contralateral projections between the pedunculopontine tegmental nucleus (PPN) and pallidal segments have been described in rats and monkeys [15,16,17,18,19,20,21]. The PPN is a mostly cholinergic nucleus located in the mesencephalic locomotor region, within the mesopontine tegmentum [22], sharing connections with the basal ganglia, thalamus, cerebral cortex and other brainstem regions [23]. Several pieces of evidence support its role in the pathophysiology of motor, cognitive and sleep-related symptoms in Parkinson’s Disease (PD) [24]. Moreover, it has been designed as an experimental target in deep brain stimulation (DBS) in PD patients with gait disturbances and postural imbalance [25].

Cholinergic innervation of the GP has also been investigated in humans, by means of immunohistochemistry techniques, suggesting that these projections are likely to ascend from the PPN [26]. Connectivity profiles of PPN have been recently reconstructed, both in monkeys and in humans, by means of diffusion tractography, a commonly used technique for probing white matter structure in vivo [27,28,29,30,31], on relatively small samples [32,33,34,35]. 

The present study is aimed at providing a detailed anatomical characterization of the pallidotegmental pathway in vivo and non-invasively via dMRI and tractography on a cohort of 100 healthy subjects from the Human Connectome Project (HCP). Specifically, we reconstructed both ipsilateral and contralateral connections of the PPN with the GP, as described in the literature on non-human primates. The number of streamlines of the pallidotegmental tracts was assessed in order to obtain a quantitative estimate of tractography results. Finally, we investigated the topographical organization of pallidotegmental connections in respect to limbic, associative and sensorimotor pallidal territories.

## 2. Materials and Methods

### 2.1. Subjects and Data Acquisition

High quality structural and dMRI data of 100 healthy subjects (males = 46, females = 54 age range 22–36 years) were collected from the HCP repository in minimally pre-processed form [36]. Data were acquired by the Washington University, University of Minnesota, and Oxford University (WU-Minn) HCP Consortium [37]. All subjects were scanned using a Siemens 3T Skyra scanner previously modified with a Siemens SC72 gradient coil and a stronger gradient power supply, with a maximum gradient amplitude (Gmax) of 100 mT/m (initially 70 mT/m and 84 mT/m in the pilot phase), with the aim of improving diffusion imaging. Diffusion-weighted images (DWI) were acquired using a single-shot 2D spin-echo multiband Echo Planar Imaging (EPI) sequence and equally distributed over 3 shells (*b*-values of 1000 s/mm^2^, 2000 s/mm^2^ and 3000 s/mm^2^), with an isotropic spatial resolution of 1.25 mm. [38]. The structural scans included T1-weighted acquisitions with the following parameters: echo time (TE) = 2.14 ms, time repetition (TR) = 2400 ms, voxel size = 0.7 mm. [39].

### 2.2. MRI Images Post-Processing

Structural images underwent brain extraction [40] and cortical and subcortical segmentation [41,42] by using BET, FAST and FIRST tools provided by FMRIB Software Library (FSL) (www.fsl.fmrib.ox.ac.uk), respectively [43]. Masks of grey matter (GM), white matter (WM) and cerebrospinal fluid (CSF) were visually inspected and, if needed, modified by a trained neuroanatomist (D.M.) in order to rule out erroneous voxel assignation, which could affect response function estimation. Once 5-tissue segmented images (5TT) were obtained, tissue-specific response functions were estimated. Finally, such response functions were used for multi-shell multi-tissue constrained-spherical deconvolution (MSMT-CSD). CSD is a signal modeling technique which allows the estimation of the fiber orientation distribution function (fODF) directly from the deconvolution of the diffusion-weighted signal with reference to a single-fiber response function [44,45]. The MSMT-CSD modeling technique represents a further implementation designed to support multi-shell data and to overcome classical CSD limitations related to fODF estimation in the presence of tissue type heterogeneity [46]. Estimation of fODFs and tractography were performed using MRtrix software (www.mrtrix.org) [47].

### 2.3. Tractography Analysis

Ipsilateral and contralateral pallidotegmental pathways were reconstructed using regions of interest (ROIs) obtained from manual segmentation and freely available atlases registered on standard Montreal Neurological Institute (MNI) space.

**Figure 1:**
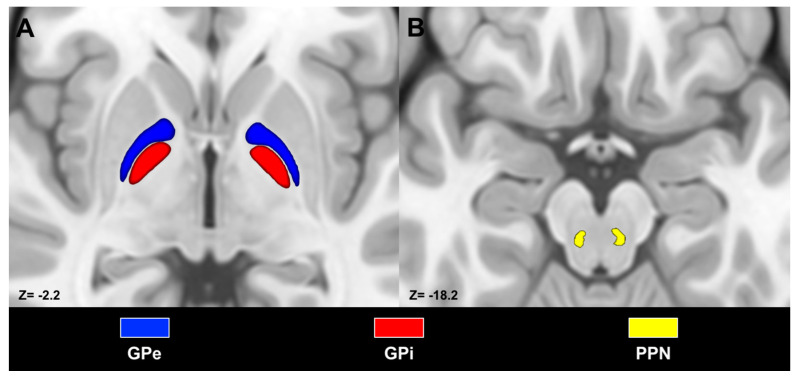
Axial sections depicting masks of pallidal segments (**A**) and pedunculopontine nucleus (**B**) superimposed on the International Consortium for Brain Mapping (ICBM) template. Masks were labeled according to the subsequent color code: external globus pallidus (GPe) (blue), internal globus pallidus (GPi) (red), pedunculopontine nucleus (PPN) (yellow). Images are shown according to radiological convention.

T1-weighed images were registered to the International Consortium of Brain Mapping (ICBM) 2009b nonlinear asymmetric template [48] employing advanced normalization tools (ANTs), specifically, symmetric diffeomorphic image registration (SyN). This registration step allowed us to obtain direct and inverse transformations [49,50]. We used inverse transformations to register the caudate, putamen, nucleus accumbens, GPi, GPe, subthalamic nucleus (STN), substantia nigra pars reticulata (SNr) and compacta (SNc) ROIs from the CIT168 Reinforcement Learning Atlas [51] and the PPN from the atlas provided by [52] to the native space of each subject (Figure 1). A rectangular mask was manually delineated on a sagittal slice at the level of the interhemispheric fissure on the ICBM 2009b nonlinear asymmetric template. This ROI corresponded to the entire extent of the medial surface of the hemispheres. The resulting mask was then registered to each subject’s native space using inverse transformations and was used as a region of avoidance (ROA) to prevent tracking of streamlines crossing the midline when ipsilateral pathways were reconstructed (see below). All the aforementioned ROIs were visually inspected and, if needed, manually modified for each subject by one of the authors (D.M.).

Ipsilateral pallidotegmental tractography was performed using pallidal ROIs as seed regions, whereas PPN ROIs were selected as included regions. When tracts were seeded from the GPi, the GPe was used as a region of avoidance (ROAs) (-exclude option of tckgen command in MRtrix3) and vice versa. Probabilistic maps of the caudate, putamen, accumbens, STN, SNc and SNr were used as ROAs to select only streamlines joining pallidal segments to the PPN. Contralateral tracts were ruled out using the abovementioned interhemispheric mask as a ROA. For contralateral pallidotegmental tractographic reconstruction, we used the GPi as a seed region and the contralateral PPN as the included region, employing contralateral probabilistic maps of the caudate, putamen, nucleus accumbens, STN, SNc and SNr as excluded regions. In each case, probabilistic tractography was performed using second order integration over fiber orientation distribution (iFOD2), which employs fiber orientation distribution (FOD), represented in the spherical harmonic basis and steps along a path given by an arc of a circle of fixed length (step size), applying a second-order probabilistic integration strategy. Overall, this algorithm allows for better characterization of streamlines with high curvatures [53]. The following tracking parameters were applied: 1000 seeds per voxel, step 1.25 × voxel size, angle 30° × step/voxel size, cutoff 0.025. These parameters have been demonstrated to perform efficient streamline tractography for tracts characterized by complex paths [54]. The number of streamlines (NOS) of the pallidotegmental tracts was then extracted in order to obtain a quantitative estimate of tractography results. Statistical analysis was carried out performing a non-parametric two-sided Wilcoxon signed rank test implemented in MATLAB 2020a in order to assess significant differences between NOS values. Left and right tracts, ipsilateral and contralateral tracts and connections of PPN to GPi and GPe were tested for statistically significant differences. Significance was set at *p* < 0.05.

### 2.4. Maximum Probability Maps and Spatial Relations with Pallidal Functional Territories

In order to provide group-level results of the pallidotegmental tracts, we sought to obtain maximum probability maps (MPMs) from track density-weighted maps reconstructed at the subject-level. We first used track data as a form of contrast to obtain track-density weighted maps of ipsilateral and contralateral pallidotegmental tracts [55]. Since track-density weighted maps retain the same header information, dimensions and voxels size of structural scans that have been used as templates, direct transformations obtained from SyN were applied to register such maps to the ICBM template. Once all track-density weighted maps were registered to a common space, they were binarized and then summed in order to obtain MPMs. These MPMs underwent a threshold of 50% in order to retain only voxels overlapping in at least half of the sample [56,57].

In addition, we sought to characterize the spatial relations between the pallidotegmental tracts and pallidal limbic, associative and sensorimotor territories. PPN connectivity clusters were obtained by implementing the following steps:

1- Streamlines of the pallidotegmental tract were mapped to voxels, thus reconstructing track density-weighted maps [58], which were then multiplied for pallidal ROIs in order to obtain pallidal voxels connected to the PPN at the subject-level.
2- An arbitrary threshold of 25% was applied to rule out voxels characterized by low track density, as in previous works [8,59].
3- Thresholded maps were registered in the ICBM template by means of direct transformations obtained by SyN.
4- Maps at the subject-level registered on the ICBM template were binarized and summed in order to obtain average maps representative of the whole sample. Furthermore, in this case, a threshold of 50% was applied to MPMs in order to retain only voxels overlapping by at least 50%.

Pallidal functional territories were obtained from a recent study by our group, conducted on the same 100 unrelated subjects of the HCP repository [60], in which we parcellated the GPi and GPe according to their main subcortical connectional systems: striatopallidal, subthalamopallidal and pallidothalamic pathways. Herein, we selected striatopallidal connectivity maps for three main reasons: (i) both GPi and GPe are connected to the striatum, allowing us to use the same parcellation as in [61]; (ii) a tripartite topographic organization was also recognized for the striatopallidal pathway in post-mortem human specimens [62]; (iii) tractography-derived parcellation of GP according to striatopallidal connectivity was already carried out in other studies with similar methodologies, gaining similar results [7]. These maps were obtained via a two-stage parcellation process: firstly, striatal (caudate, putamen and nucleus accumbens) parcellation was carried out according to its connectivity to four cortical targets representing limbic, associative sensorimotor and “other” territories [7,8,63]. Once striatal parcellation was carried out, the resulting connectivity clusters were used to perform parcellation of the GPi and GPe according to striatopallidal connectivity.

Finally, we calculated the percentage of overlapping voxels between the PPN pallidal maps and maps of pallidal functional territories derived from GP parcellation based on striatopallidal connectivity. The degree of overlap between PPN pallidal maps and pallidal functional territories was measured as a percentage overlap, which is defined as [64]:

(1) $$\%\mathrm{overlap}=\frac{A\;\cap^{\;}B}{B}\times 100$$

where *A* and *B* correspond to the volume (measured in voxels) of the two thresholded MPMs. In our case, *A* represents PPN thresholded MPMs, whereas *B* stands for thresholded MPMs of pallidal functional territories.

## 3. Results

### 3.1. Anatomical Characterization of the Pallidotegmental Tract

**Figure 2:**
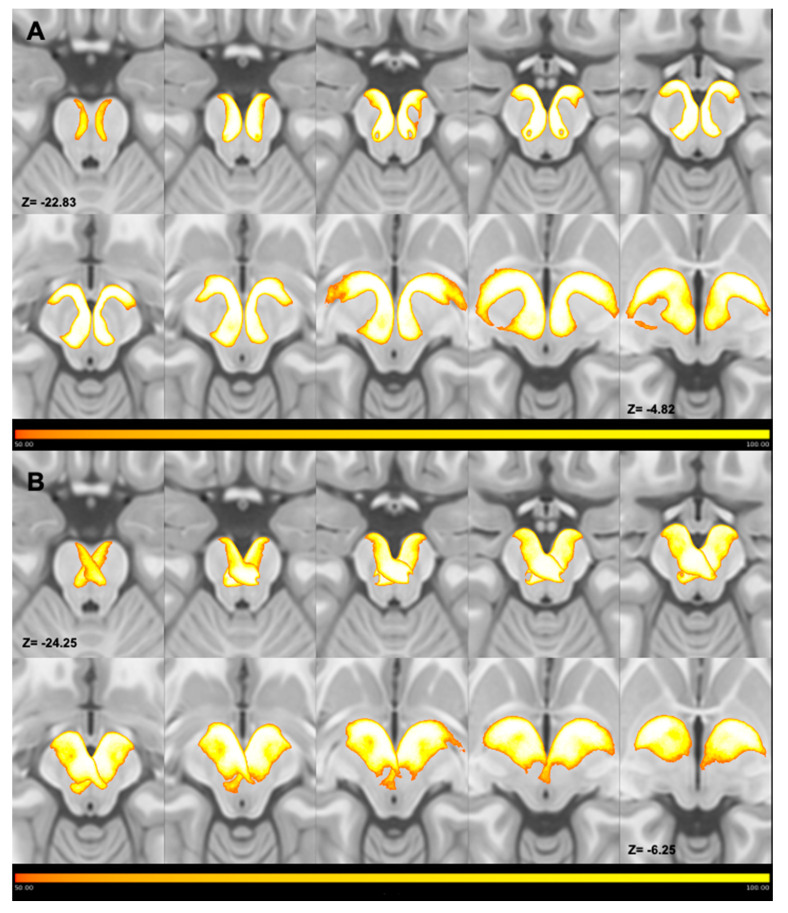
Multiple axial slices showing the course of thresholded maximum probability maps (MPMs) of ipsilateral (**A**) and contralateral (**B**) tracts joining the PPN and GPi. Ipsilateral and contralateral MPMs have been superimposed on the ICBM template and displayed in the form of a lightbox, which shows the course of the average maps in a caudal-to-cranial direction. As shown by color bars, the voxel intensity is proportional to the number of subjects; thus, voxels characterized by lowest intensities overlapped in no more than 50 subjects, whereas voxels characterized by highest intensities overlapped across all subjects. Ipsilateral maps traverse the central tegmentum near the midline in the most caudal slices, travel in proximity to the red nucleus and, in the most cranial slices, enter the cerebral peduncle, finally reaching the GPi. Contralateral maps cross the midline at the mesencephalic decussation, as shown in the most caudal slices, and reach the contralateral GPi after entering the cerebral peduncle. Images are shown according to radiological convention.

Tracts joining the PPN and both pallidal segments were successfully reconstructed in all subjects (*n* = 100) and showed an overall similar anatomical course, which was well-summarized by the MPMs (Figure 2). The pallidotegmental tract, joining the PPN with the ipsilateral GPi, ran through the medial portion of the central mesencephalic tegmentum, passing above the red nucleus, then entering the cerebral peduncle and finally reaching the GPi (Figure 2A). The pallidotegmental tract connecting the PPN to the contralateral GPi traversed the mesopontine tegmentum, crossing the midline at the level of the mesopontine tegmentum decussation and finally entered the GPi (Figure 2B).

**Figure 3:**
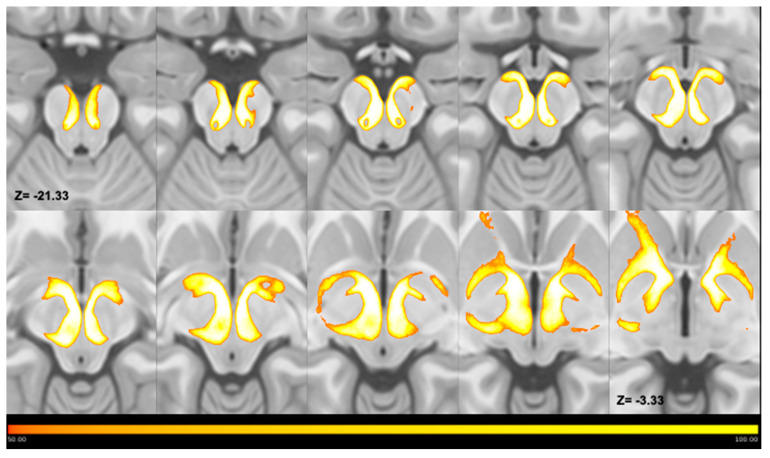
Multiple axial slices showing the course of thresholded MPMs of tracts joining the PPN and GPe. MPMs have been superimposed on the ICBM template and displayed in the form of a lightbox, which shows the course of the average maps in a caudal-to-cranial direction. As shown by color bars, the voxel intensity is proportional to the number of subjects; thus, voxels characterized by lowest intensities overlapped in no more than 50 subjects, whereas voxels characterized by highest intensities overlapped in all subjects. Maps joining the PPN to the GPe ascend through the mesencephalic tegmentum in the most caudal slices and reach the GPe after entering the cerebral peduncle in the most cranial slices. Left and Right are reported according to radiological convention.

The pallidotegmental tract between the PPN and the GPe traversed the midbrain tegmentum, progressively ascending in its medial portion, reaching the pallidal complex, where, after bending in close proximity to the GPi anterior pole, reached the anterior ventral aspect of the GPe (Figure 3). In line with the available anatomical evidence, we did not reconstruct crossed connections between the GPe and PPN, since their presence has been neglected in primate studies [21].

### 3.2. Quantitative Analysis

The quantitative analysis (mean ± standard error) revealed higher NOS in the GPi compared to the GPe (left GPi–ipsilateral PPN 807.7 ± 26.7, left GPe–ipsilateral PPN 368.6 ± 12.0, *p* < 0.001; right GPi–ipsilateral PPN 1024.7 ± 35.3, right GPe–ipsilateral PPN 547.8 ± 16.4, *p* < 0.001).

Moreover, left contralateral pallidotegmental tracts exhibited higher NOS values when compared to left ipsilateral tracts (left GPi–ipsilateral PPN 807.7 ± 26.7, left GPi–contralateral PPN 990.4 ± 31.1, *p* < 0.01). On the other hand, right ipsilateral pallidotegmental tracts showed higher NOS values than right crossed ones (right GPi–ipsilateral PPN 1024.7 ± 35.3, right GPi–contralateral PPN 895.5 ± 31.8, *p* < 0.001). 

Finally, significant left/right differences were observed among ipsilateral pallidotegmental tracts; specifically, NOS values of tracts joining the PPN with both the GPi (left GPi–ipsilateral PPN 807.7 ± 26.7, right GPi–ipsilateral PPN 1024.7 ± 35.3, *p* < 0.001) and the GPe (left GPe–ipsilateral PPN 368.6 ± 12.0, right GPe–ipsilateral PPN 547.8 ± 16.4, *p* < 0.001) resulted to be significantly right-lateralized. Finally, left/right differences were also observed for contralateral pallidotegmental tracts (left GPi–contralateral PPN 990.4 ± 31.1, right GPi–contralateral PPN 895.5 ± 31.8, *p* < 0.01).

### 3.3. Spatial Relation with Pallidal Territories

**Figure 4:**
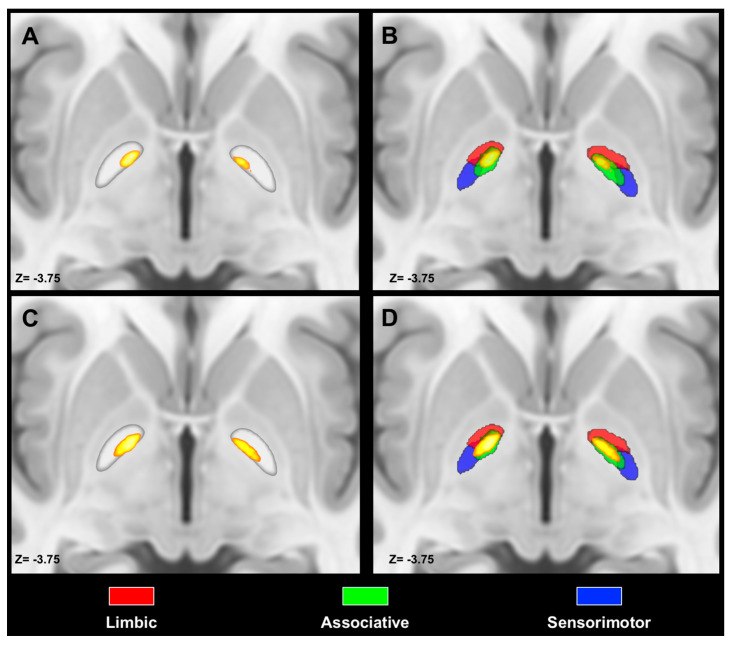
Spatial relations between PPN MPMs and the GPi. Axial slices of the ICBM template showing ipsilateral (**A**,**B**) and contralateral (**C**,**D**) PPN MPMs are superimposed as heatmaps on both the globus pallidus regions of interest (ROIs) and the pallidal limbic, sensorimotor and functional territories. Images are shown according to radiological conventions.

**Figure 5:**
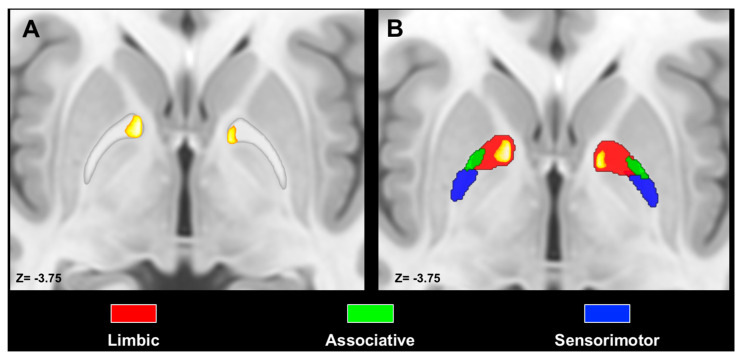
Spatial relations between PPN MPMs and the GPe. Axial slices of the ICBM template showing PPN MPMs superimposed on both the globus pallidus ROIs (**A**) and the pallidal limbic, associative and sensorimotor territories (**B**). Images are shown according to radiological conventions.

We observed notable differences between the GPi and GPe concerning the topographic organization of PPN MPMs. Indeed, we observed that PPN MPMs were localized in the ventromedial aspect of the GPi, whereas they occupied the anterior pole and the most ventral portion of the GPe. Spatial arrangement between ipsilateral PPN MPMs and tractography-derived MPMs of pallidal functional territories unveiled the highest values of percentage overlap for associative territories (left: 28%; right 31%), followed by limbic (left: 25%, right 26%) and sensorimotor (left 0.2%; right 5%). The same pattern was observed for the contralateral PPN–GPi tracts, with the highest percentage overlap exhibited by associative (left: 39%, right 42%) territories, followed by limbic (left: 27%, right 30%) and sensorimotor territories (left 5%, right 15%) (Figure 4). Conversely, percentage overlap was less represented for the GPe, with the highest values for limbic (left: 9%; right 21%), followed by associative territories (left 7%; right 5%); no overlap was observed between PPN MPMs and sensorimotor territories in the GPe (Figure 5).

## 4. Discussion

The present study was aimed at reconstructing in vivo and non-invasively the structural connections between pallidal segments and the PPN, in order to provide an anatomical characterization of ipsilateral and contralateral pallidotegmental tracts. Indeed, although existing studies conducted in humans via dMRI tractography have explored PPN connectivity profiles at the whole-brain level, here we focused only on the connections with pallidal segments. To the best of our knowledge, the abovementioned studies did not investigate differences between the GPi and GPe when studying pallidotegmental connectivity, considering the globus pallidus as a whole. Herein, we characterized, for the first time, the topographical organization of the pallidotegmental pathways within the GPi and GPe and their spatial relations with respect to pallidal functional territories.

We successfully reconstructed pallidotegmental pathways already described in non-human primates [16,21,65] and also in humans [32,35,66]. To the best of our knowledge, this is the first time that the crossed pallidotegmental pathway between the PPN and GPi has been reconstructed in vivo in humans by means of tractography. We found that both the ipsilateral and contralateral pallidotegmental bundles here reconstructed followed an anatomical course similar to those described in primates [16,21]. Ipsilateral tracts traversed the medial portion of mesopontine tegmentum, ascending through the cerebral peduncle and finally reaching the ipsilateral pallidal complex [21]. During their course, such tracts were in close spatial relationship with the red nucleus, in line with previous observations on primates which hypothesized a synaptic interaction with the magnocellular part of the red nucleus [65]. However, it is important to keep in mind that tractography does not allow inferences at such a dimensional scale (see the Limitations section), thus this observation remains to be confirmed and more studies are necessary to evaluate the relationship between the human red nucleus and pallidotegmental tracts in light of notable inter-species differences in red nucleus structural connectivity [59].

Contralateral pallidotegmental pathways crossed the midline mainly at the level of the superior cerebellar peduncle decussation, reaching the contralateral GPi by passing through the cerebral peduncles, similarly to the description provided in [16]. Although being already described in monkeys, contralateral pallidotegmental tracts constitute a novel finding in humans. Even if PPN functional anatomy is likely to be similar in monkeys and humans [33,67], such findings need to be further confirmed by other studies.

NOS values revealed that streamlines joining the PPN to the GPi are significantly more represented than those connecting the PPN to the GPe, in line with primate studies which described only “few scattered cells” labelled in the PPN after injection of tracers in the GPe [15]. 

The higher NOS between the GPi and the PPN is apparently in contrast with a recent study conducted on squirrel monkeys which revealed that cholinergic innervation of the GPe is much more pronounced than in the GPi [68]. This mismatch may be explained by the fact that the PPN is not the only source of cholinergic innervation of pallidal segments. Indeed, studies of immunoreactivity for the nerve growth factor receptors (NGF-R) suggested that, although in the human GPi cholinergic afferents arise mainly from the PPN, those of the GPe are likely to originate from the nucleus basalis of Meynert [26,69]. Another possible explanation of this finding relies on the heterogenous chemical anatomy of the PPN, which includes not only cholinergic neurons but also glutamatergic and GABAergic neurons [70]. Indeed, it has also been hypothesized that non-cholinergic neurons populations (especially GABAergic neurons) may project into the globus pallidus [23]. Finally, a recent study combining tract tracing in monkeys and tractography in humans [35] showed that PPN connectivity is higher with the GPi than with the GPe.

Our quantitative analysis showed that the NOS of contralateral tracts were significantly higher than ipsilateral ones. This is surprising as existing studies conducted on monkeys reported that the contralateral pathway was less represented when compared to the ipsilateral pallidotegmental tract [21]. As the contralateral pallidotegmental tract has been poorly investigated in humans, this difference may be a peculiarity which marks a difference between humans and monkeys. However, due to tractography tendency to generate false positives, it is not possible to completely exclude that this difference is grounded on technical rather than biological reasons.

Similarly, the interpretation of significant left/right differences in terms of connectivity strength is not immediate. Indeed, no quantitative analysis has been performed in previous studies employing tractography to characterize in vivo PPN connections [32,33,34], except for the study conducted by Sebillé and colleagues where the connectivity of the PPN and the cuneiform nucleus were compared using a percentage of connected voxels from the target nuclei [35]. However, left/right differences were not evaluated in that study. Even if some lateralization has been noticed between PPN pallidal territories when investigating the topography of PPN connections in monkeys [71], no clear explanatory hypothesis has been provided. Thus, the functional meaning of such lateralization is unclear, and more studies are needed to further elucidate this subject.

When investigating the topography of PPN connectivity maps, we found a good correspondence between the structural connectivity-based topography of the pallidotegmental tract and that provided by primate studies. Indeed, it has been argued that, in non-human primates, axons of PPN neurons reaching the GPi and GPe show a different spatial organization. In particular, fibers arborizing in the GPi are more abundant in its ventromedial aspect, whereas those reaching the GPe cluster more ventrally, avoiding its central portion [16]. In line with non-human primate studies, we showed that PPN clusters are located in the ventromedial portion of GPi probabilistic maps, whereas they occupy mainly an anteroventral position within the GPe maps. Moreover, examining the spatial relations with limbic, associative and sensorimotor pallidal territories, we found that pallidotegmental maps are mainly localized within the associative territory in the GPi, while being situated within the limbic clusters in the GPe. This result matches well with studies assessing the functional topography of the globus pallidus in primates [72,73], as also reported in a recent review concerning pallidal afferent connections in the brainstem [14]. Taken together, the results of the present study support the hypothesis that, despite being connected to both pallidal segments, pallidotegmental connections may exert a different modulatory role in the GPi and the GPe [15]. However, more studies are needed to better understand the physiological, behavioral and even pathophysiological underpinnings of such differences in structural connectivity.

### 4.1. Pathophysiological and Therapeutic Implications

The role of the PPN and its surrounding structures in locomotion has been studied for decades in cats and rats [74,75,76] and has been corroborated by more recent studies conducted in non-human primates and humans [77,78,79]. Additionally, a well-represented body of literature supports the influence of PPN cholinergic and non-cholinergic neurons in the regulation of sleep/wake cycles; specifically, PPN neurons exhibit higher firing rates during wake and REM sleep, suggesting a role in determining arousal-like states [23].

PPN involvement in the pathophysiology of motor and non-motor symptoms of PD has been the subject of several investigations [24,80,81]. Several findings have suggested that degeneration of PPN neurons in patients with PD may be related to both motor and non-motor symptoms [24,70,82]. As a consequence of neuronal degeneration, pallidotegmental pathways may be affected in PD, resulting in a loss of cholinergic inputs to both the GPi and the GPe [14]. It has been hypothesized that pallidal loss in acetylcholine may induce non-motor symptoms such as attention deficit and stereotyped behaviors [83,84,85,86]. Even if the results here presented have been obtained from a sample of healthy subjects, they may foster future research evaluating how microstructural properties and metrics of fiber density differ among healthy subjects, PD patients without gait disturbances and PD patients with impaired locomotion and postural instability. Moreover, it would be noteworthy to look for differences in pallidotegmental tracts between patients with Parkinson’s dementia and patients who do not develop cognitive complications.

Among gait disorders commonly observed in PD, freezing of gait (FOG), defined as a sudden inability to move forward during gait initiation or during walking, constitutes the most emblematic and, at the same time, the least understood [87]. Although the exact mechanism behind FOG or different FOG subtypes is far from being elucidated, no neuronal loss in the PPN has been noticed in 1-methyl-4-phenyl-1,2,3,6-tetrahydropyridine (MPTP) animal models, abandoning the idea of a direct role of the PPN in FOG [88]. On the other hand, results of neuroimaging studies conducted on humans proposed that the PPN plays a compensatory role in patients with FOG. According to this hypothesis, FOG is likely to appear when such compensation fails [22]. To the best of our knowledge no studies have yet investigated the structural connectivity between the GP and the PPN in patients with FOG. Hence, tractographic reconstruction of pallidotegmental tracts may be used to quantify connectivity strength or to measure microstructural properties in this peculiar subset of patients, in order to provide a deeper understanding of FOG in PD.

The functional properties of the PPN and its role in the pathophysiology of a wide spectrum of PD symptoms have led to a growing interest in PPN stimulation in patients unresponsive to canonical pharmacological and surgical treatments [25]. The first attempts at PPN DBS targeting in PD patients with refractory gait disorders date back to the early 2000s [89,90], and results of DBS studies conducted over the years have been recently reviewed [91]. The overall consensus is that stimulation of the PPN in patients with gait disorders has a beneficial effect, reducing FOG episodes and falls [25]. The exact mechanism through which PPN-area stimulation is likely to ameliorate gait disorders in PD patients is still unclear. However, it may be related to: (i) the activation of neuronal populations projecting into central pattern generation of the spinal cord, (ii) the influence on the basal ganglia and cerebellum, (iii) the activation of the reticular activating system and/or (iv) the activation of the medial lemniscus and disruption of pathological oscillation in the cerebral cortex [22]. The pallidotegmental tracts here reconstructed may represent one of the white matter tracts through which PPN DBS exerts its beneficial effects in PD patients [92]. New approaches, allowing the isolation of bundles whose stimulation is associated with and may predict better clinical outcomes, may be suitable to further elucidate therapeutic implications of pallidotegmental tracts in future studies on patient cohorts [93,94]. It is important to keep in mind that PPN targeting is challenging due to its small size, position and the heterogeneity of the mesencephalic locomotor area. For these reasons, PPN DBS may lead to valuable side effects [91,95]. Hence, further studies on large patient cohorts are required to better clarify the advantages and the related risks of PPN targeting. On the other hand, we believe that DBS of the PPN area and its effects at a connectomic level may shed new light on treatment opportunities for such a hard-to-treat subset of PD patients.

### 4.2. Limitations

We acknowledge that our work suffers from several intrinsic technical limitations. Firstly, tractography allows us to obtain only indirect representations of white matter bundles, consisting of streamlines which do not match axon diameter, and it does not allow us to distinguish afferent from efferent connections [96]. Thus, we cannot distinguish between pedunculopallidal and pallidopeduncular connections, and with the term “pallidotegmental pathway” throughout the paper, we refer to both these kinds of connections, without totally excluding the possible contribution of neighboring structures belonging to the mesencephalic locomotor area (e.g., the cuneiform nucleus). Due to several weaknesses of tractography, tract-tracing techniques remain the gold standard to investigate structural connectivity [97]; however, such methodologies are not applicable in vivo in the human brain. Despite the aforementioned limitations, diffusion tractography remains the only available method to probe structural connectivity in vivo, and existing studies revealed a good correspondence between tractographic reconstruction of white matter bundles and dissection studies [98]. The reliability of tractography largely depends on data quality, diffusion signal modeling and the tracking algorithm employed. In our study, we combined high quality data from the HCP repository in order to obtain the most reliable results [99,100], along with MSMT-CSD, which prevents the estimation of fODF outside white matter [46]. In addition, although the bundles reconstructed in the present study have been already described in several non-human primates [15,20,65] and in humans as well [14,34,35], we cannot totally rule out the presence of false positives [101] due to the use of probabilistic tractography. Finally, the use of NOS as a quantitative measure is not to be interpreted as “fiber count” or “fiber density” [102]. Although some correction strategies to overcome the limitations of streamline count as a quantitative connectivity measure have been proposed [103], it has been shown that these correction methods only provide an incomplete compensation for the inherent biases of NOS [104]. Indeed, it is worth mentioning that new methods to assess streamline density more reliably have been recently proposed, even though they are computationally expensive [105,106]. However, none of the aforementioned approaches has produced a measure of connecting axons, which is desirable in neuroanatomical studies [107]. Hence, the quantitative measures of the present pilot study should be taken with a grain of salt and interpreted as a quantitative metric to describe tractography results, rather than connectivity strength. For this reason, we look forward to further widening the sample size and implementing novel and more reliable measures of connectivity density.

## 5. Conclusions

In this study, we aimed at characterizing anatomical connections joining both pallidal segments and the PPN. We reconstructed both ipsilateral and contralateral pallidotegmental tracts for the GPi and ipsilateral tracts for the GPe, using findings provided by studies conducted on monkeys as our main point of reference. Our results suggest that notable differences in PPN connectivity with the GPi and the GPe may exist in humans. Such differences concern the NOS reconstructed and the topographical organization of pallidal voxels connected to the PPN. Further studies are warranted to better understand the relevance of these differences both in healthy and diseased subjects. We hope that this study will foster new research about brainstem connections with pallidal segments in humans, further widening the interest in their modulatory role on motor and non-motor behaviors.
